# Dopamine D_2_ Receptor-Mediated Heterologous Sensitization of AC5 Requires Signalosome Assembly

**DOI:** 10.1155/2012/210324

**Published:** 2012-03-12

**Authors:** Karin F. K. Ejendal, Carmen W. Dessauer, Terence E. Hébert, Val J. Watts

**Affiliations:** ^1^Department of Medicinal Chemistry and Molecular Pharmacology, College of Pharmacy, Purdue University, 575 Stadium Mall Drive, West Lafayette, IN 47907-2051, USA; ^2^Department of Integrative Biology and Pharmacology, University of Texas Health Science Center at Houston, Houston, TX 77030, USA; ^3^Department of Pharmacology Therapeutics, McGill University, McIntyre Medical Sciences Building, Montréal, QC, Canada H3G 1Y6

## Abstract

Chronic dopamine receptor activation is implicated in several central nervous system disorders. Although acute activation of G*α*_i_-coupled D_2_ dopamine receptors inhibits adenylyl cyclase, persistent activation enhances adenylyl cyclase activity, a phenomenon called heterologous sensitization. Previous work revealed a requirement for G*α*_s_ in D_2_-induced heterologous sensitization of AC5. To elucidate the mechanism of G*α*_s_ dependency, we expressed G*α*_s_ mutants in G*α*_s_-deficient *Gnas* ^E2−/E2−^ cells. Neither G*α*_s_-palmitoylation nor G*α*_s_-G*βγ* interactions were required for sensitization of AC5. Moreover, we found that coexpressing *β*ARKct-CD8 or Sar1(H79G) blocked heterologous sensitization. These studies are consistent with a role for G*α*_s_-AC5 interactions in sensitization however, G*βγ* appears to have an indirect role in heterologous sensitization of AC5, possibly by promoting proper signalosome assembly.

## 1. Introduction 

Dopamine receptors and dopamine signaling have been implicated in various neurological and psychiatric disorders including Parkinson's disease, schizophrenia, and drug abuse [1–3]. Dopamine receptors are divided into two subfamilies, the G*α*_s_-coupled D_1_ and D_5_ receptors and the G*α*_i/o_-coupled D_2_, D_3_, and D_4_ dopamine receptors that have stimulatory and inhibitory effects on adenylyl cyclase (AC), respectively (see [3] for a recent review). Acute stimulation of D_2_ dopamine receptors leads to inhibition of AC activity, however, persistent activation of this G*α*_i/o_-coupled receptor paradoxically results in its enhancement [4]. This phenomenon, called heterologous sensitization of AC, is also known as cAMP overshoot, supersensitization, or superactivation of AC. D_2_ dopamine receptor-induced heterologous sensitization of cyclic AMP signaling has been demonstrated in several cellular systems as well as in animal models and has also been suggested to occur in humans [4–6]. For example, it was observed that repeated administration of the D_2_ receptor agonist quinpirole enhances AC activity in the caudate putamen, increases CREB phosphorylation, and also alters behavior in rodents [5, 6]. Although this mode of AC regulation has been recognized for over three decades [7], the molecular signaling mechanism causing heterologous sensitization of AC is only partially understood, attributed to some extent to differences in AC isoform-specific regulation [4].

There are nine differentially regulated membrane-bound AC isoforms in mammalian cells [4, 8]. Whereas all AC isoforms are stimulated by stimulatory G*α*_s_, only a subset is inhibited by inhibitory G*α*_i_, and some AC isoforms are differentially regulated by G*βγ* [4, 8]. Here, we studied human adenylyl cyclase type 5 (AC5) that is potently stimulated by G*α*_s_, inhibited by acute activation of G*α*_i_, and conditionally activated by G*βγ* [8]. AC5 is expressed at high levels in the central nervous system and has been identified as a primary effector of D_2_ dopamine receptors in the striatum [9, 10].

The aim of the current study was to investigate the role(s) of heterotrimeric G proteins in D_2_ receptor-mediated heterologous sensitization of AC5. By exploring sensitization in cells devoid of endogenous G*α*_s_ [11], we were able to examine the ability of G*α*_s_ mutants to support sensitization without interference from endogenous G*α*_s_. Additionally, this G*α*_s_-deficient cellular model expresses very low levels of AC5 making them a reasonable model for studies of recombinant AC5 [12]. Heterologous sensitization of AC5 was readily rescued by wild-type G*α*_s_ and by mutants deficient in palmitoylation [13] or G*βγ* interaction [14]. We also assessed the role of G*βγ* and the signalosome in D_2_ receptor-induced heterologous sensitization of AC5 by sequestering G*βγ* subunits with *β*ARKct-CD8 [15, 16] and coexpressing a dominant-negative mutant of the Sar1 GTPase [17]. These experiments revealed that both *β*ARKct-CD8 and Sar1(H79G) attenuated sensitization, suggesting that the components of the signaling complex utilized in heterologous sensitization, presumably AC5 and G*α*_s_, assemble postsynthesis in the endoplasmic reticulum (ER). Together with previous findings, the present data support a model in which G*α*_s_ directly interacts with AC5. In contrast, G*βγ* appears to have an indirect role in heterologous sensitization of AC5.

## 2. Materials and Methods

### 2.1. Constructs

The human D_2L_ receptor and AC5 or ΔAC5 [18] were cloned into the dual expression vector pBUDCE4 (Invitrogen, Carlsbad, CA) creating pBUD/hAC5, D_2_R and pBUD/ΔAC5, D_2_R. pcDNA3/*β*ARKct-CD8 [15, 16] and pcDNA/vsvg-Sar1 (wild type and H79G) [19] were used. pcDNA1/G*α*_s_-CFP [20] was a gift from Dr. Catherine Berlot. The pcDNA3.1/G*α*_s_-IEK+ mutant [21] was a gift from Dr. Philip Wedegaertner. The C3S mutation was created by site-directed mutagenesis, and the fragment containing the IEK+ mutations was amplified by PCR. The resulting constructs, pcDNA1/G*α*_s_-CFP(C3S) and pcDNA1/G*α*_s_-CFP(IEK+) were sequenced.

### 2.2. Cell Culture and Transient Transfection

All reagents were purchased from Sigma-Aldrich (St. Louis, MO) unless otherwise noted. G*α*_s_-deficient murine embryonic fibroblast cells, *Gnas* ^E2−/E2−^ cells [11, 12], were a gift from Dr. Murat Bastepe. Cells were cultured in 50 : 50 mix of F12 : DMEM media supplemented with 5% FBS (HyClone, Logan, UT), 1% Ant-Anti (Invitrogen, Carlsbad, CA) in a humidified incubator at 33°C with 5% CO_2_. Approximately 80,000 cells/well were seeded in 24-well plates the day before transient transfection. DNA (400 ng pBUD/hAC5 or ΔAC5, D_2_R alone or in combination with 10 ng pcDNA/G*α*_s_-CFP, 300 ng pcDNA3/*β*ARKct-CD8, or 300 ng pcDNA/vsvg-Sar1) was mixed with Opti-MEM and 1 *μ*L/well Lipofectamine 2000 (Invitrogen, Carlsbad, CA). The medium was replaced with 200 *μ*L/well prewarmed Opti-MEM, and the DNA/Lipofectamine mixture was added to the cells. After 4 hr, culture medium (500 *μ*L/well) was added, and the cells were analyzed after 48 hr. For microscopy, the amount of pcDNA/G*α*_s_-CFP was increased to 100 ng/well. 

### 2.3. Acute cAMP Accumulation

The assays were carried out in assay buffer (EBSS supplemented with 0.2% ascorbic acid, 15 mM HEPES, and 2% BCS (HyClone, Logan, UT), and 500 *μ*M IBMX) with 100 nM forskolin (Tocris Bioscience, Ellisville, MO) as noted for 37°C for 15 min. The media was decanted, ice-cold trichloroacetic acid was added, and the lysates were stored at 4°C. Cyclic AMP was quantified using a competitive binding assay as described previously [22]. Data were collected from a minimum of three independent experiments carried out in duplicate and were normalized to either basal or vehicle conditions. The GraphPad Prism 5 software (GraphPad Software Inc., LaJolla, CA) was used for data and statistical analyses. A *P* value of ≤0.05 was considered statistically significant.

### 2.4. Heterologous Sensitization

The cells were pretreated with 1 *μ*M quinpirole or vehicle in assay buffer (without IBMX) for 2 hr followed by three washes. cAMP was measured as described above for acute cAMP accumulation, with the addition of 1 *μ*M spiperone to block the action of any residual quinpirole.

### 2.5. Microscopy

Cells were seeded in cover glass slides (Nunc, Rochester, NY). A 12 bit photometric CoolSNAP (Roper Scientific) CCD camera mounted on a TE-2000 inverted epifluorescence microscope (Nikon Instruments Inc., Melville, NY) with filters (ex. 500/20, em. 535/30) from Chroma (Rockingham, VT) was used. Images were acquired with the MetaMorph software (Molecular Devices, Sunnyvale, CA) and analyzed using Image J (http://rsbweb.nih.gov/ij/).

## 3. Results and Discussion

### 3.1. G*α*_s_ Mutants Rescue Heterologous Sensitization of AC5

**Figure 1:**
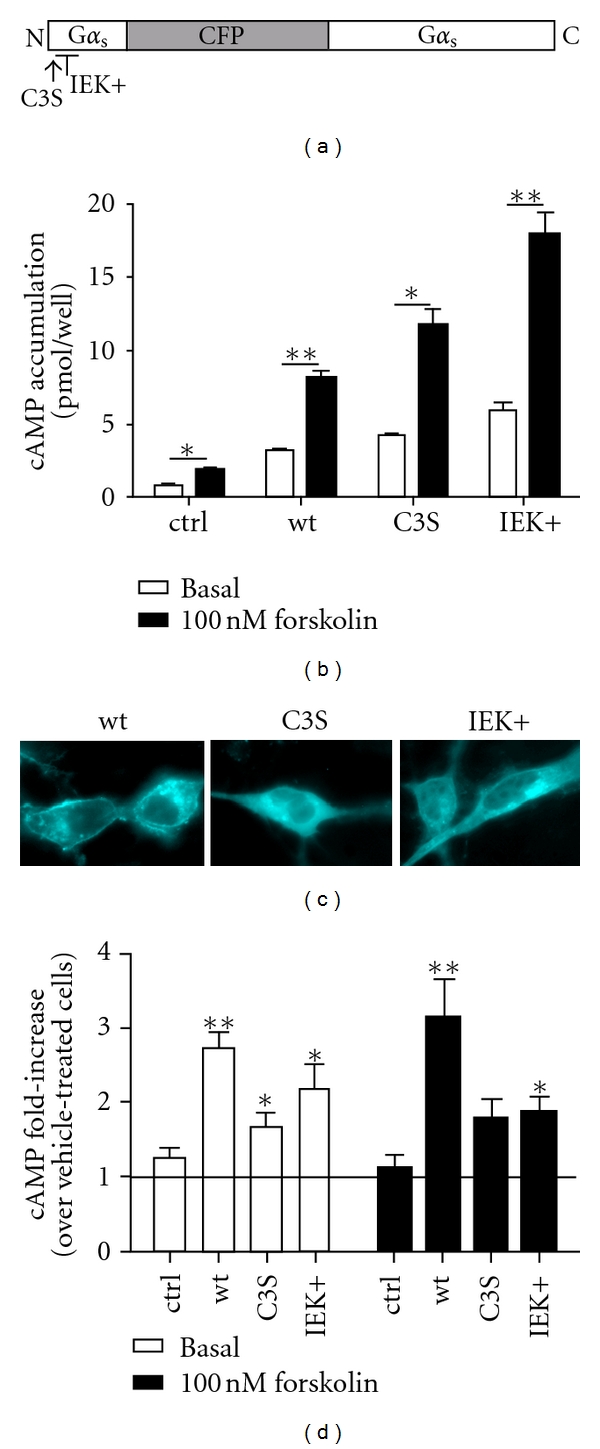
G*α*_s_-CFP mutants are functional and rescue heterologous sensitization of AC5. (a) Schematic of G*α*_s_-CFP constructs. (b) Acute cAMP accumulation in cells expressing AC5 and D_2_ alone (ctrl) or in combination with 10 ng G*α*_s_-CFP (wild type, C3S, or IEK+) was measured under basal (open bars) or forskolin-stimulated conditions (black bars). **= *P* < 0.01, *= *P* < 0.05, using a paired, one-tailed *t*-test comparing basal and forskolin-stimulated values. (c) Expression and localization of G*α*_s_-CFP mutants. (d) Heterologous sensitization of AC5 in cells expressing AC5 and D_2_ in the absence or presence of G*α*_s_-CFP. Data shown represent fold-increase of cAMP accumulation observed in quinpirole-treated cells. **= *P* < 0.01, *= *P* < 0.05, using a one-sample, two-tailed *t*-test comparing ctrl to each G*α*_s_-CFP construct.

Our laboratory has previously shown that mutants of canine AC5 that do not interact with G*α*_s_ are deficient in sensitization [23, 24] and that D_2_-mediated heterologous sensitization of AC5 has an absolute requirement for G*α*_s_ [12]. Our present objective was to elucidate the mechanism of G*α*_s_-dependent heterologous sensitization of human AC5 by utilizing two different G*α*_s_-CFP [20] mutants (Figure 1(a)). The C3S substitution eliminates the N-terminal palmitoylation site, which causes G*α*_s_ to mislocalize to the cytosolic fraction [13]. The IEK+ mutant contains a series of substitutions, yielding a G*βγ*-binding deficient G*α*_s_ that also displays a reduction in palmitoylation [21].

The G*α*_s_-CFP constructs were coexpressed with AC5 and D_2_. Since both C3S and IEK+ are deficient in responses to receptor stimulation [13, 21], we used direct stimulation of AC5 with forskolin throughout this study. Basal cAMP accumulation without any G*α*_s_ was 0.73 ± 0.09 pmol/well, whereas co-expression of G*α*_s_-CFP increased cAMP accumulation to 3.12 ± 0.22 pmol/well (wild-type, wt), 4.22 ± 0.06 pmol/well (C3S), and 5.88 ± 0.05 pmol/well (IEK+). Forskolin further stimulated cAMP with values 2.5–3-fold over basal levels (Figure 1(b)), indicating that wild-type and both G*α*_s_ mutants functionally couple to AC5. Next, expression and subcellular localization of the G*α*_s_-CFP constructs (in the presence of AC5 and D_2_) were evaluated by fluorescence microscopy (Figure 1(c)). Wild-type G*α*_s_-CFP showed both plasma membrane and intracellular localization, whereas the C3S and IEK+ mutants were predominantly localized intracellularly (Figure 1(c)), consistent with previous reports [14, 21].

To assess whether the G*α*_s_-CFP mutants could rescue heterologous sensitization, cells were pretreated with vehicle or quinpirole followed by cAMP accumulation. Consistent with our previous report [12], no sensitization of AC5 was observed in the absence of G*α*_s_ (Figure 1(d), ctrl). In contrast, coexpression of wild-type G*α*_s_-CFP resulted in robust sensitization of AC5 under both basal and forskolin-stimulated conditions (Figure 1(d)). Surprisingly, expression of the G*α*_s_ mutants also significantly rescued heterologous sensitization under basal conditions (white bars) and to a lesser degree forskolin-stimulated conditions (black bars). As both mutants are deficient in palmitoylation and membrane localization, neither palmitoylation *per se*, nor membrane localization of G*α*_s_ appears to be essential for heterologous sensitization of AC5.

### 3.2. Role of G*βγ* Subunits in Heterologous Sensitization of AC5

**Figure 2:**
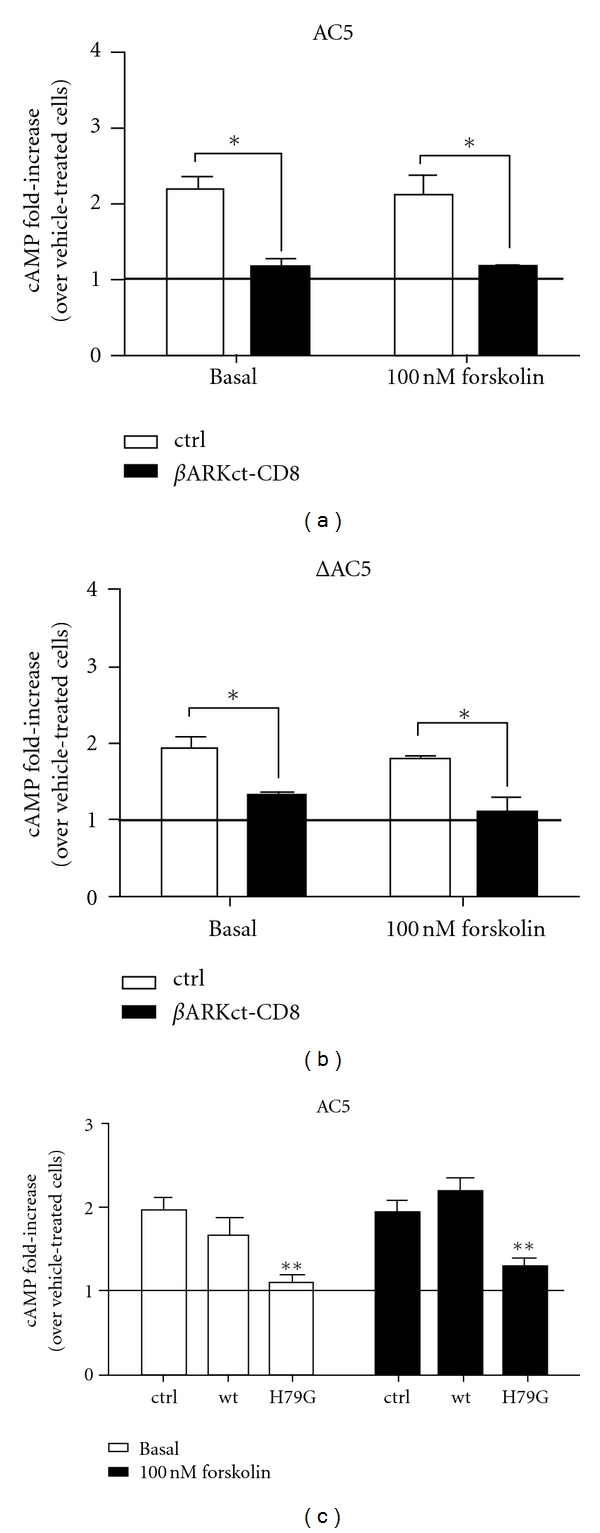
Sequestration of G*βγ* with *β*ARKct-CD8 or coexpression of dominant negative Sar1(H79G) attenuate heterologous sensitization of AC5. Cells expressing G*α*_s_-CFP, D_2_R and (a and c) AC5 or (b) ΔAC5 in combination with either empty vector (ctrl), (a-b) *β*ARKct-CD8, or (c) indicated Sar1 construct. Data shown represent the fold-increase of cAMP accumulation observed in quinpirole-treated cells. (a-b) *= *P* < 0.05, using a paired, one-tailed *t*-test. (c) **=*P* < 0.01, using a one-way ANOVA and Dunnett's *post hoc* test, comparing ctrl to Sar1(wt) or Sar1(H79G).

Although we have established that G*α*_s_ is required for heterologous sensitization, our findings above for the IEK+ mutant suggest that direct interactions between G*α*_s_ and G*βγ* are not critical. This prompted us to further investigate the role of G*βγ* in D_2_ receptor-mediated heterologous sensitization of AC5. The C-terminus of *β*-adrenergic kinase or GRK2 (*β*ARKct) has been used to sequester G*βγ* subunits and inhibit G*βγ*-mediated signaling events, including heterologous sensitization [15, 25, 26]. In the absence of *β*ARKct-CD8 (membrane bound *β*ARKct), AC5 displayed robust heterologous sensitization (open bars, Figure 2(a)). Sequestering G*βγ* blocked sensitization of AC5, under both basal and forskolin-stimulated conditions, revealing the necessity of G*βγ* for heterologous sensitization of AC5 (black bars, Figure 2(a)). In contrast, *β*ARKct-CD8 had no substantial effects on acute D_2_ receptor activation; quinpirole produced significant inhibition of cAMP accumulation in the presence of *β*ARKct-CD8 (77 ± 10% inhibition; *n* = 2, data not shown). In an effort to explore the site of action for G*βγ*-dependent sensitization, we used an N-terminal deletion mutant of AC5, ΔAC5. This mutant is functional and responds to G*α*_s_ stimulation but is deficient in binding G*βγ* [18]. The ΔAC5 mutant displayed significant sensitization that was also blocked by *β*ARKct-CD8 (Figure 2(b)), suggesting that N-terminal G*βγ* binding is not intimately involved in heterologous sensitization of AC5. Instead, there are clearly additional, unidentified G*βγ* interaction sites in AC5 that are necessary for heterologous sensitization. Such an assumption is supported by FRET and *in vitro* activation studies of the AC5 deletion mutant [18] as well as studies of AC2, which possesses multiple motifs for G*βγ* interaction and regulation that are located in the C1b and C2b domains of AC2 [27]. Other possibilities are that ΔAC5 interacts with endogenous AC isoforms in an AC dimer (see [28]) that binds G*βγ* or that specific G*β* and G*γ* subunits or G*βγ* pairs are involved. However, it is also possible that the G*βγ* mechanisms involving sensitization of AC may be indirect [4].

### 3.3. Disruption of Signalosome Assembly Affects Heterologous Sensitization of AC5

Because sequestering G*βγ* subunits alters signalosome assembly [15], we hypothesized that a specific signaling complex could be required for heterologous sensitization of AC5. Several small GTPases, including Sar1, are involved in signal complex assembly and anterograde protein trafficking [29]. A series of studies using dominant negative mutants of these GTPases shows that G*α*_s_ and G*βγ* interact with AC2 during trafficking to the plasma membrane [30, 31] and that the G*α*_s_-AC2 interaction is disrupted by Sar1(H79G) [30].

To study the possibility that interactions between AC5 and its specific signaling partners play a role, we utilized Sar1 and Sar1(H79G) and noted that coexpression with the dominant negative mutant prevented heterologous sensitization of AC5 (Figure 2(c)). In contrast, acute D_2_ receptor-mediated inhibition of AC5 was not significantly blocked in the presence of Sar1(H79G) (data not shown). Our data are consistent with the findings that Sar1(H79G) disrupts AC-G*α*_s_ interactions (as measured by BRET or coimmunoprecipitation) to a larger degree than AC-G*α*_i_ interactions [30]. In contrast, Sar1(H79G) did not affect the interactions between AC and G*βγ* [30], suggesting that the AC interacts with G*βγ* at an early step in the endoplasmic reticulum (ER), but that the interaction with G*α*_s_ occurs after ER export. The observation that signaling mechanisms of acute activity and heterologous sensitization are differentially affected further supports the hypothesis that heterologous sensitization and acute stimulation are dependent on separate mechanisms and possibly separate signalosome components.

## 4. Conclusion

The present data support a complex model of D_2_ dopamine receptor-induced heterologous sensitization of AC5 where G*α*_s_ appears to directly interact with AC5. A role for G*βγ* was confirmed; however, our observations suggest an indirect role for G*βγ* that may be involved during the formation of the sensitization signaling complex. A critical role for AC5 in mediating dopamine responses has been previously demonstrated in AC5 deficient mice, which show impaired responses to D_2_ receptor activation [9]. Therefore, these results have implications in brain regions where D_2_ dopamine receptors and AC5 are coexpressed, such as the striatum [32], which is implicated in drug addiction, motivation, mood, and voluntary movement. Persistent D_2_ dopamine receptor activation has also been linked to psychiatric disorders (e.g., schizophrenia and drug abuse) and to the adaptive responses associated with drug therapy in Parkinson's disease. Enhancing our understanding of the underlying components and mechanisms of heterologous sensitization and regulation of specific AC activity (in the striatum) may aid in the development of improved and future therapies for these disorders. For example, recent studies have identified small molecule inhibitors of G*βγ*-mediated signaling [33] and AC isoform-specific inhibitors [34] that may offer novel therapeutic strategies for modulating complex CNS behaviors involving dopamine receptor signaling.

## Abbreviations

AC: Adenylyl cyclase
cAMP: Cyclic adenosine monophosphate
CFP: Cyan fluorescent protein
D_2_R: Dopamine D_2L_ receptor
GPCR: G protein-coupled receptor
*β*ARKct: *β*-adrenergic kinase c-terminus
ER: Endoplasmic reticulum
